# Effects of Mepiquat Chloride Application Timing and Concentration on Soybean Plant Architecture and Stem Mechanical Properties

**DOI:** 10.3390/plants15203126

**Published:** 2026-10-13

**Authors:** Jiaqi Li, Zhipeng Qu, Xiaomei Li, Shenghan Du, Yuchen Zhou, Boyang Li, Shoukun Dong

**Affiliations:** 1 College of Agriculture, Northeast Agricultural University, Harbin 150030, China; 2 Hulunbuir Agricultural Technology Extension Center, Hulunbuir 021008, China; 3 College of Agriculture, Heilongjiang Agricultural Engineering Vocational and Technical University, Harbin 150025, China

**Keywords:** cell wall components, mepiquat chloride, plant architecture, soybean, stem mechanical properties

## Abstract

Soybean plant architecture and stem mechanical properties are important determinants of lodging-resistance potential. To clarify the regulatory effects of mepiquat chloride (MC) applied at different growth stages and concentrations on soybean stem traits, two cultivars, Heinong 44 (HN44) and Heinong 65 (HN65), were used. Water-sprayed plants served as the control (MCK), and MC was applied foliarly at 50, 100, 200, or 400 mg/L at the V3 and R1 stages. Plant height, stem diameter, stem mechanical traits, and cell-wall components were measured. The effects of MC were strongly dependent on application timing, concentration, and cultivar. Following V3 application, 400 mg/L MC reduced the plant height of HN44 by 21.85% at R1; 200 mg/L MC increased basal stem diameter by 15.01% at R3 and fifth-internode diameter by 17.94% at R4. In HN65, 400 mg/L MC increased bending deflection by 27.01% at R1, whereas 100 mg/L MC increased cellulose content by 36.50%. In HN44, 100 mg/L MC increased lignin content by 77.39% at R2. Following R1 application, 400 mg/L MC increased bending deflection of HN44 by 29.40% at R2; 50 mg/L MC increased basal stem diameter by 20.85% at R6; and 400 mg/L MC increased stem breaking force by 36.23% at R4. In HN65, 200 mg/L MC increased hemicellulose content by 42.62% at R6. Correlation analysis showed a significant positive correlation between hemicellulose and lignin after both V3 and R1 applications, while some cell wall components were negatively correlated with stem bending deflection or breaking force. Overall, MC applied at different growth stages and concentrations regulated soybean plant architecture, stem mechanical properties, and cell wall composition; however, no single concentration was optimal across all cultivars and traits. V3 application produced a more pronounced early effect on plant architecture.

## 1. Introduction

Soybean (*Glycine max* (L.) Merr.) is an important global food and oil crop and a major source of plant protein, with broad applications in food, feed, and bioenergy production [1,2]. With increasing planting density in soybean production and the growing frequency of extreme wind and rain events, lodging has become an important constraint on high and stable yields and mechanized harvesting [3]. The effects of lodging on yield vary among growth stages, with lodging at early flowering and early seed filling causing particularly severe yield losses [4,5]. Lodging not only disrupts canopy structure and reduces radiation-use efficiency, but also complicates mechanical harvesting and decreases yield and seed quality [6]. Therefore, improving soybean lodging-resistance potential by optimizing plant architecture, lowering the plant center of gravity, and enhancing stem mechanical strength is an important objective in high-yield soybean cultivation.

Mepiquat chloride (MC) is a systemic plant growth retardant that can be absorbed by leaves and roots and translocated within the plant. One of its major effects is the inhibition of cell elongation, thereby regulating longitudinal plant growth [7]. Because MC is effective at relatively low doses and leaves comparatively little residue, it has been widely used to regulate plant architecture in various crops [8]. Previous studies have shown that MC can significantly reduce plant height, and in crops such as cotton, its inhibitory effect generally becomes stronger as application concentration increases [9]. The reduction in plant height is mainly associated with suppressed internode elongation. Moderate internode shortening can lower the plant center of gravity, improve canopy structure, reduce mutual shading, and increase canopy radiation transmittance, thereby contributing to greater lodging resistance [10]. However, the effects of MC are influenced by crop species, genotype, growth stage, and application concentration; consequently, the appropriate application stage and concentration for soybean remain unclear.

In addition to morphological traits such as plant height and stem diameter, stem mechanical properties are important indicators of crop lodging resistance. Stem breaking force and bending deflection reflect the ability of a stem to withstand external mechanical failure and deformation [11]. Previous studies have shown that soybean stem mechanical strength is closely associated with stem morphology, and excessive stem elongation and reduced stem diameter are usually accompanied by decreases in stem strength and lodging resistance [12]. Previous studies [13] reported close relationships between soybean lodging resistance and stem breaking force and bending deflection, with higher values generally indicating greater resistance to mechanical damage. Kamran et al. [14] further showed that MC treatment increased maize stem puncture strength, bending performance, and stem diameter, thereby improving stem mechanical properties. Stem mechanical performance is determined not only by external morphology but also by cell-wall structure and composition. Plant cell walls provide the major mechanical support for stems, and lignin, cellulose, and hemicellulose are key structural constituents that determine cell-wall strength and toughness [15,16]. Lignin deposition increases cell-wall rigidity and stem load-bearing capacity [17]; cellulose microfibrils help maintain cell-wall strength and stability [18,19]; and hemicellulose interacts with cellulose and lignin through complex cross-linking structures, jointly influencing cell-wall mechanical properties [20]. Accordingly, MC regulation of stem mechanical properties may involve not only morphological changes in plant height and stem diameter but also redistribution or accumulation of structural cell-wall components.

Although previous studies have shown that MC can regulate plant architecture and improve stem mechanical performance in several crops, research in soybean has largely focused on a single growth stage or a single concentration. The combined regulatory effects of application timing and concentration on soybean plant architecture, stem mechanical properties, and cell-wall components remain poorly understood, and systematic comparisons of cultivar-specific responses are still limited. Our previous study showed that HN44 and HN65 exhibited markedly different responses to MC treatment in terms of stem mechanical properties and lodging resistance-related traits. The two cultivars differed in the response patterns of stem breaking force, bending characteristics, and lodging resistance index to MC concentration and treatment duration [21]. Based on these findings, HN44 and HN65 were selected for the present study to further compare the effects of MC applied at different growth stages and concentrations on plant architecture, stem mechanical properties, and cell wall components between the two cultivars. Plant height, stem diameter, stem breaking force, bending deflection, and cellulose, hemicellulose, and lignin contents were evaluated at multiple developmental stages. This study therefore examined whether the regulatory effects of MC depend on application stage, concentration, and cultivar and whether these effects are associated with changes in stem cell wall components. The objective was to identify suitable MC application timings and concentrations and provide a theoretical basis for the chemical regulation of soybean plant architecture and lodging resistance.

## 2. Results

### 2.1. Effects of MC Application Timing and Concentration on Soybean Plant Height

**Figure 1:**
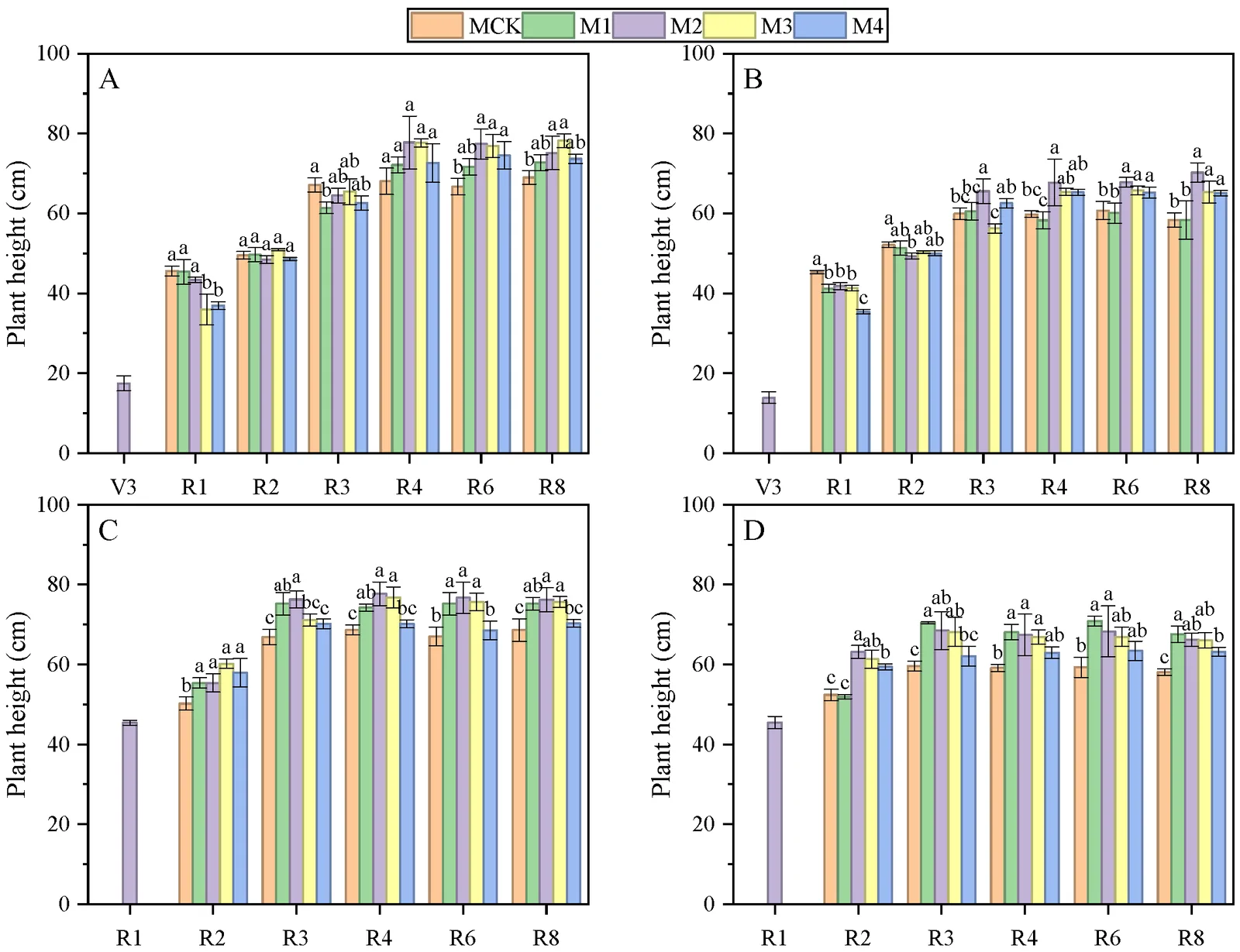
Effects of MC application stage and concentration on soybean plant height. (**A**), HN65 treated at V3; (**B**), HN44 treated at V3; (**C**), HN65 treated at R1; (**D**), HN44 treated at R1. MCK, M1, M2, M3, and M4 represent 0, 50, 100, 200, and 400 mg/L MC, respectively. Different lowercase letters indicate significant differences among MC concentration treatments within the same cultivar and sampling stage.

As shown in Figure 1, soybean plant height responded differently to MC depending on application timing, concentration, developmental stage, and cultivar. Following V3 application, some MC treatments reduced plant height during the early post-treatment period, but this effect gradually weakened as development progressed. In contrast, MC applied at R1 did not produce sustained height suppression, and concentration-dependent responses diverged during the middle and late growth stages.

Following V3 application, plant height of both HN65 and HN44 was generally lower than MCK at R1. In HN65, M1, M2, M3, and M4 reduced plant height by 0.37%, 4.83%, 21.08%, and 18.96%, respectively, relative to MCK, with larger numerical reductions under M3 and M4. The corresponding reductions in HN44 were 9.05%, 7.73%, 8.90%, and 21.85%, with the greatest decrease under M4. As development progressed, differences among treatments gradually narrowed, and plant height under some MC treatments reached or exceeded MCK during the middle and late growth stages. In HN44 at R8, plant height under M2 and M3 was 20.34% and 12.00% higher than MCK, respectively, indicating that suppression of longitudinal growth by V3 treatment was concentrated mainly in the early period after application. Following R1 application, neither cultivar showed the sustained suppression observed after V3 application. In HN65 at R2, plant height under all MC treatments was higher than MCK, with M3 increasing it by 20.00%. In HN44 at R2, M2 and M3 increased plant height by 20.53% and 17.09%, respectively, relative to MCK. Thereafter, treatment differences varied with growth stage, and some concentrations continued to maintain relatively greater plant height.

### 2.2. Effects of MC Application Timing and Concentration on Soybean Basal Stem Diameter

**Figure 2:**
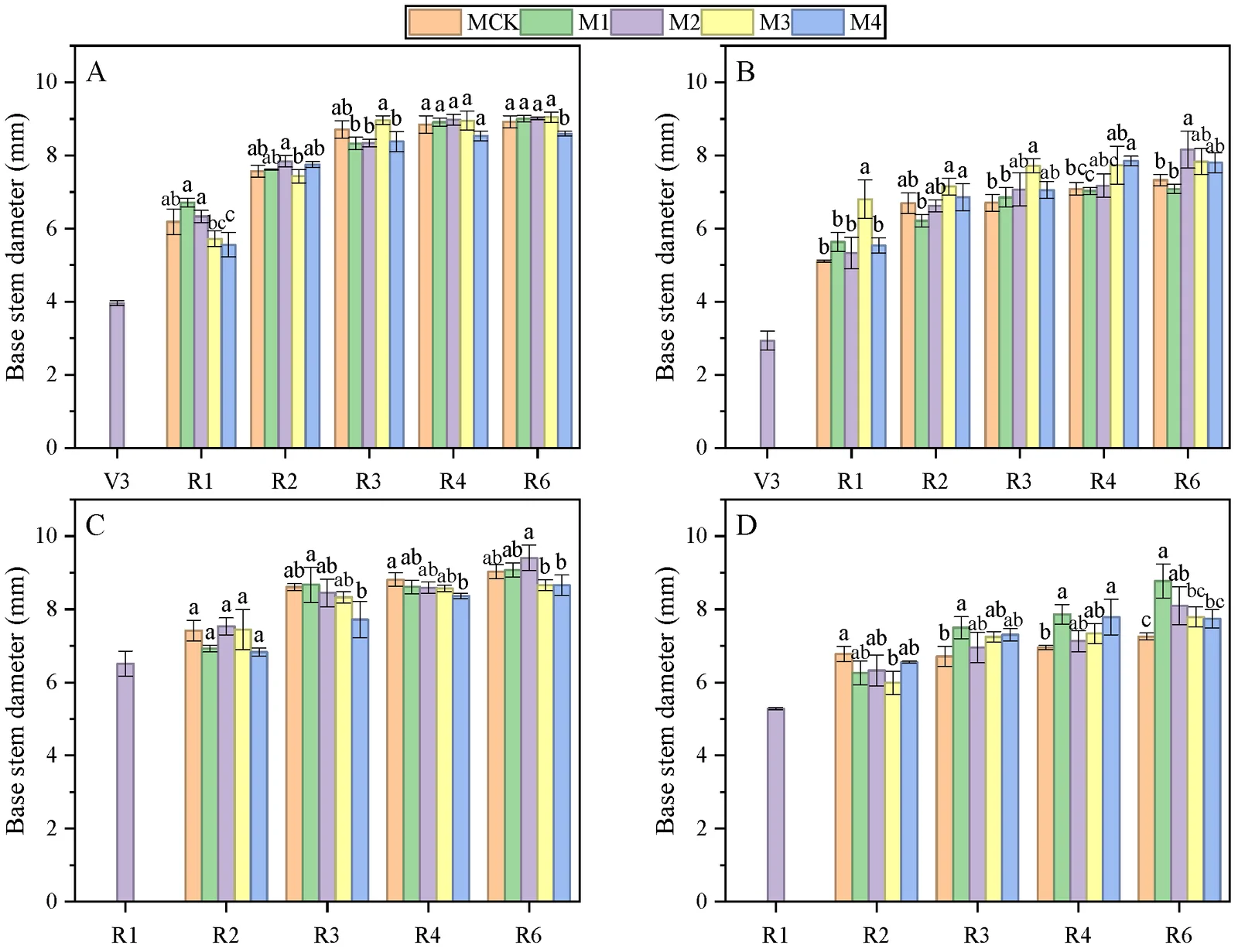
Effects of MC application stage and concentration on soybean basal stem diameter. (**A**), HN65 treated at V3; (**B**), HN44 treated at V3; (**C**), HN65 treated at R1; (**D**), HN44 treated at R1. MCK, M1, M2, M3, and M4 represent 0, 50, 100, 200, and 400 mg/L MC, respectively. Different lowercase letters indicate significant differences among MC concentration treatments within the same cultivar and sampling stage.

As shown in Figure 2, basal stem diameter varied with MC application stage and concentration. The promotive effect of MC on basal stem diameter was relatively limited in HN65, whereas HN44 showed more pronounced stem thickening at several developmental stages, particularly during the middle and late growth periods.

Following V3 application, HN65 showed stimulation at lower concentrations but inhibition at higher concentrations at R1. Relative to MCK, basal stem diameter increased by 8.52% and 2.43% under M1 and M2 but decreased by 7.44% and 10.08% under M3 and M4, respectively. By R6, treatment differences from MCK were small: M1, M2, and M3 increased basal stem diameter by only 0.97%, 1.05%, and 1.42%, respectively, whereas M4 decreased it by 3.66%. The response of HN44 was more pronounced. At R3, M3 increased basal stem diameter by 15.01% relative to MCK; at R6, M1 was slightly lower than MCK, whereas M2, M3, and M4 increased basal stem diameter by 11.38%, 6.92%, and 6.60%, respectively, indicating that appropriate MC concentrations promoted radial stem growth in HN44. Following R1 application, cultivar responses diverged further. In HN65 at R6, M2 increased basal stem diameter by 4.13% relative to MCK, whereas M3 and M4 reduced it by 4.13% and 4.17%, respectively, indicating a relatively weak overall thickening effect. In HN44 at R6, all MC treatments exceeded MCK, with increases of 20.85%, 11.58%, 7.30%, and 6.61% under M1, M2, M3, and M4, respectively; M1 produced the largest increase.

### 2.3. Effects of MC Application Timing and Concentration on the Fifth-Internode Diameter of Soybean

**Figure 3:**
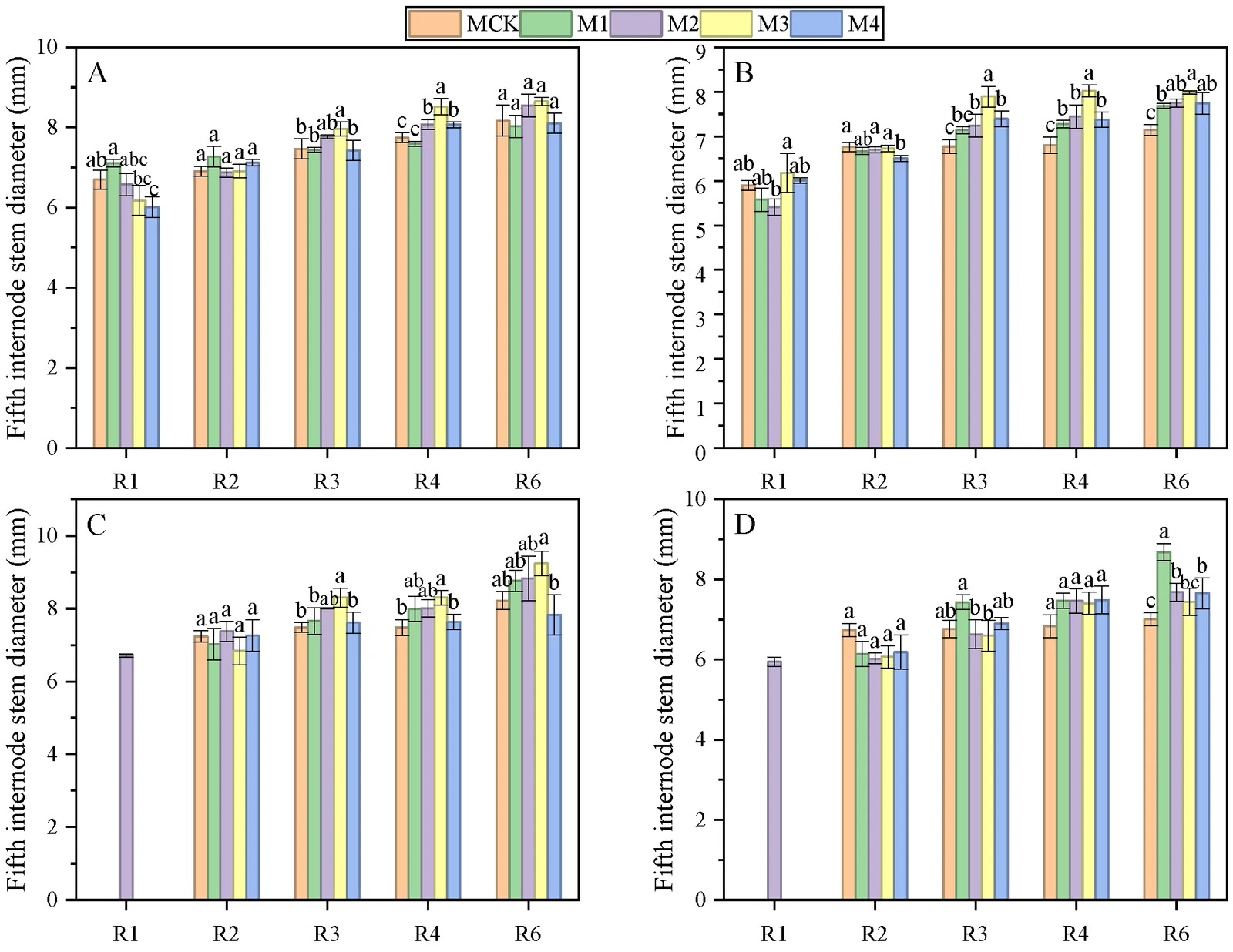
Effects of MC application stage and concentration on soybean fifth-internode diameter. (**A**), HN65 treated at V3; (**B**), HN44 treated at V3; (**C**), HN65 treated at R1; (**D**), HN44 treated at R1. MCK, M1, M2, M3, and M4 represent 0, 50, 100, 200, and 400 mg/L MC, respectively. Different lowercase letters indicate significant differences among MC concentration treatments within the same cultivar and sampling stage.

As shown in Figure 3, MC application stage and concentration affected fifth-internode diameter, with responses varying by concentration, developmental stage, and cultivar. A concentration of 200 mg/L produced a relatively strong thickening effect at several growth stages, and the response of HN44 was comparatively stable during the middle and late stages.

Following V3 application, HN65 showed clear treatment differences at R1, R3, and R4; from R3 to R6, fifth-internode diameter generally increased and then decreased as MC concentration increased. At R6, M1 and M3 produced larger fifth-internode diameters than MCK. The response of HN44 to V3 application was more pronounced: from R2 to R6, the 200 mg/L treatment generally produced relatively large diameters, and at R4, M3 increased fifth-internode diameter by 17.94% relative to MCK. At R6, M1, M2, M3, and M4 increased the diameter by 7.61%, 8.54%, 11.81%, and 8.50%, respectively, with the largest increase again under M3. Following R1 application, HN65 generally exhibited an increase followed by a decrease in fifth-internode diameter with increasing MC concentration at R3, R4, and R6. M3 increased fifth-internode diameter by 11.01% at R3 and 10.91% at R4 relative to MCK, representing the largest increase at both stages. In HN44, all MC treatments generally produced larger fifth-internode diameters than MCK at R4 and R6; at R4, M1, M2, M3, and M4 increased the diameter by 9.26%, 9.22%, 8.34%, and 9.51%, respectively.

### 2.4. Effects of MC Application Timing and Concentration on Soybean Stem Breaking Force

**Figure 4:**
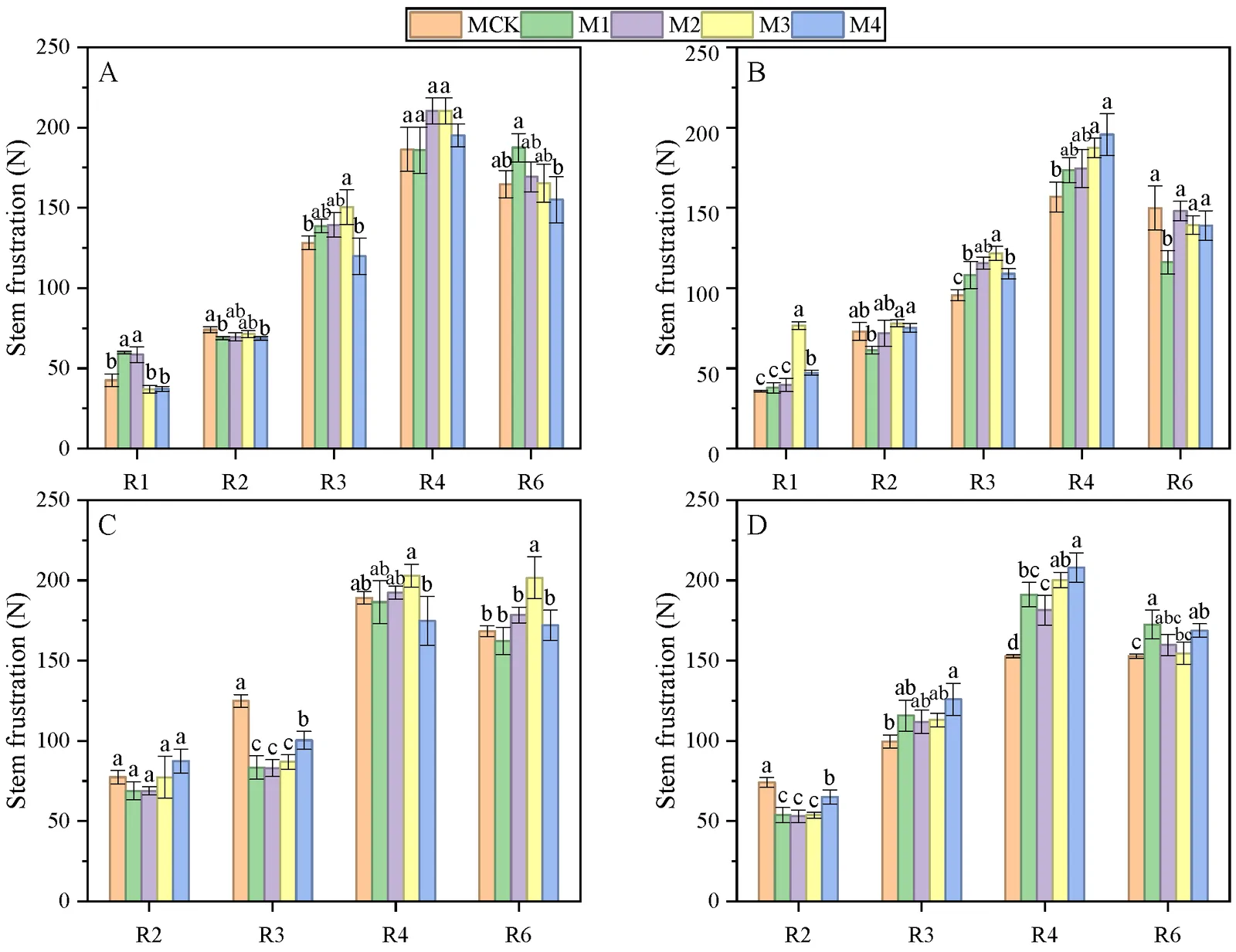
Effects of MC application stage and concentration on soybean stem breaking force. (**A**), HN65 treated at V3; (**B**), HN44 treated at V3; (**C**), HN65 treated at R1; (**D**), HN44 treated at R1. MCK, M1, M2, M3, and M4 represent 0, 50, 100, 200, and 400 mg/L MC, respectively. Different lowercase letters indicate significant differences among MC concentration treatments within the same cultivar and sampling stage.

As shown in Figure 4, stem breaking force varied with MC treatment and growth stage. In both cultivars, stem breaking force generally increased and then decreased as plant development progressed, with relatively high values observed at the R4 stage.

Following V3 application, both cultivars showed clear concentration responses during the early treatment period. In HN65 at R1, stem breaking force under M1 and M2 was 40.34% and 37.53% higher than MCK, respectively, whereas the advantage of the higher-concentration treatments was less pronounced. In HN44 at R1, all MC treatments exceeded MCK, with increases of 5.75%, 10.52%, 113.64%, and 31.63% under M1, M2, M3, and M4, respectively; M3 produced the largest increase. As development progressed, breaking force increased rapidly across treatments and reached high levels at R4 before declining by R6, indicating that the effect of MC on mechanical strength was also modulated by plant development. Following R1 application, both cultivars also exhibited relatively high stem breaking force at R4. In HN65, the 400 mg/L treatment performed relatively well at R4 and R6. In HN44 at R4, all MC treatments exceeded MCK, and M4 increased breaking force by 36.23%. At R6, M1, M2, M3, and M4 in HN44 remained 12.97%, 4.63%, 1.26%, and 10.58% higher than MCK, respectively, indicating that the enhancement persisted into later development in some treatments.

### 2.5. Effects of MC Application Timing and Concentration on Soybean Stem Bending Deflection

**Figure 5:**
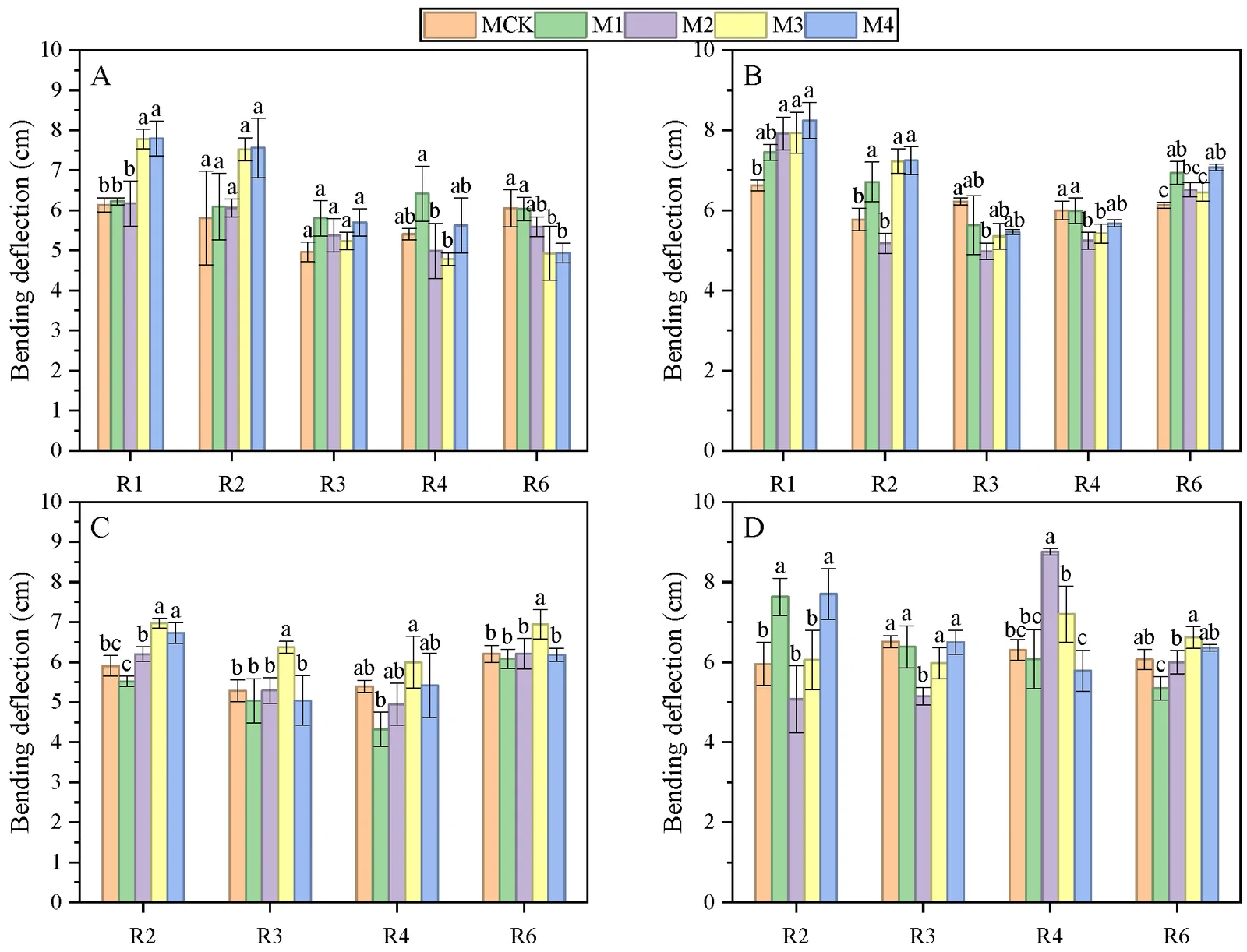
Effects of MC application stage and concentration on soybean stem bending deflection. (**A**), HN65 treated at V3; (**B**), HN44 treated at V3; (**C**), HN65 treated at R1; (**D**), HN44 treated at R1. MCK, M1, M2, M3, and M4 represent 0, 50, 100, 200, and 400 mg/L MC, respectively. Different lowercase letters indicate significant differences among MC concentration treatments within the same cultivar and sampling stage.

Bending deflection responded to MC but did not increase monotonically with concentration; instead, it varied markedly with developmental stage and cultivar (Figure 5). Compared with stem breaking force, bending deflection showed greater fluctuation in its concentration response across sampling stages.

Following V3 application, bending deflection of both cultivars was generally higher than MCK at R1. In HN65, M1, M2, M3, and M4 increased bending deflection by 1.52%, 0.60%, 26.85%, and 27.01%, respectively, with larger increases under M3 and M4. At R3, the corresponding increases were still 17.08%, 8.54%, 5.65%, and 14.93%. In HN44 at R1, M1, M2, M3, and M4 increased bending deflection by 12.43%, 19.58%, 19.83%, and 24.46%, respectively, indicating that V3 MC treatment markedly altered early stem bending-deformation characteristics in both cultivars. Following R1 application, the concentration responses of the two cultivars diverged further. In HN65 at R2, M1, M2, M3, and M4 changed bending deflection by −6.49%, 5.02%, 18.00%, and 13.88%, respectively, relative to MCK, with the greatest increase under M3. In HN44 at R2, the corresponding changes were 28.22%, −14.78%, 1.68%, and 29.40%, demonstrating a nonlinear response. Differences among treatments remained evident at R4 and R6.

### 2.6. Effects of V3 MC Application on Soybean Stem Cell-Wall Components

**Figure 6:**
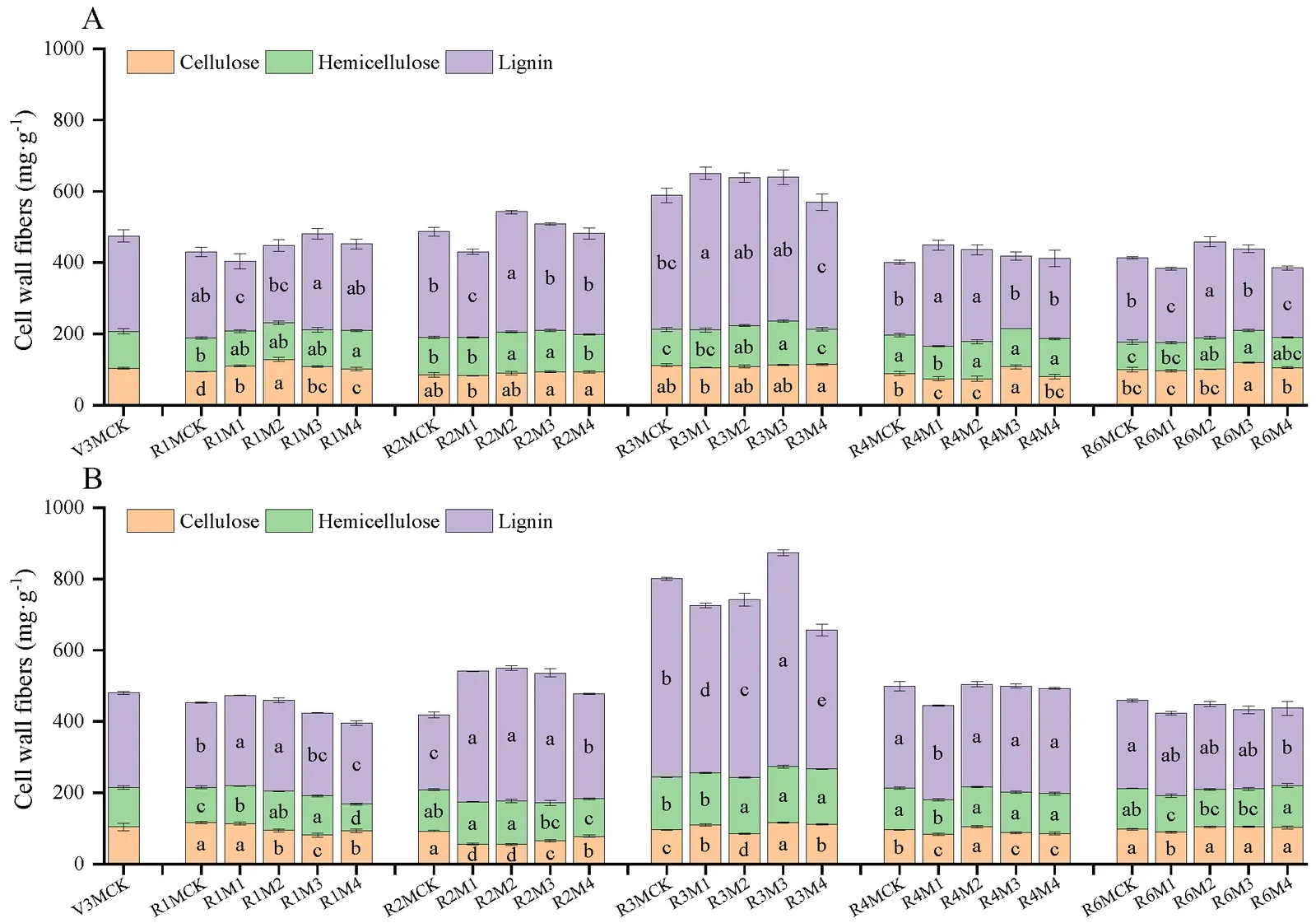
Effects of different MC concentrations applied at V3 on soybean stem cell wall components. (**A**), HN65; (**B**), HN44. V3MCK denotes the MCK treatment sampled at V3; R1MCK–R1M4, R2MCK–R2M4, R3MCK–R3M4, R4MCK–R4M4, and R6MCK–R6M4 denote treatments MCK–M4 sampled at R1, R2, R3, R4, and R6, respectively. MCK is the water-sprayed control (0 mg/L MC); M1, M2, M3, and M4 represent 50, 100, 200, and 400 mg/L MC, respectively. Different lowercase letters indicate significant differences among MC treatments for the same cell wall component within the same cultivar and sampling stage (*p* < 0.05).

As shown in Figure 6, V3 application of MC altered cellulose, hemicellulose, and lignin contents in stems of HN65 and HN44, although the concentration responses differed among components. Overall, 200 mg/L MC produced relatively pronounced regulatory effects on cell-wall components at several growth stages, whereas 400 mg/L also caused inhibition at some stages, indicating that cell-wall components did not accumulate linearly with increasing MC concentration.

HN65 showed a relatively clear cellulose response during the early period after treatment. At R1, M1, M2, M3, and M4 increased cellulose content by 17.46%, 36.50%, 15.57%, and 8.39%, respectively, relative to MCK, with the largest increase under M2. Hemicellulose generally increased with concentration at R1, and M4 was 13.05% higher than MCK; by R6, M2 and M3 increased hemicellulose by 13.70% and 16.60%, respectively. The lignin response changed with growth stage: at R2, only M2 showed a relatively clear increase, whereas at R4, M1 and M2 increased lignin by 38.79% and 26.19%, respectively, at R4, demonstrating distinct response patterns among cell wall components.

HN44 exhibited a response pattern distinct from that of HN65. At R3, cellulose content under M1, M3, and M4 was 14.92%, 21.62%, and 15.71% higher than MCK, respectively, whereas M2 reduced it by 10.92%. At R1, hemicellulose increased by 5.69%, 10.17%, and 10.73% under M1, M2, and M3, respectively, but decreased by 25.28% under M4. Lignin showed the most pronounced response to V3 MC application: at R2, M1, M2, M3, and M4 increased lignin by 74.44%, 77.39%, 73.40%, and 40.07%, respectively, relative to MCK.

### 2.7. Effects of R1 MC Application on Soybean Stem Cell-Wall Components

**Figure 7:**
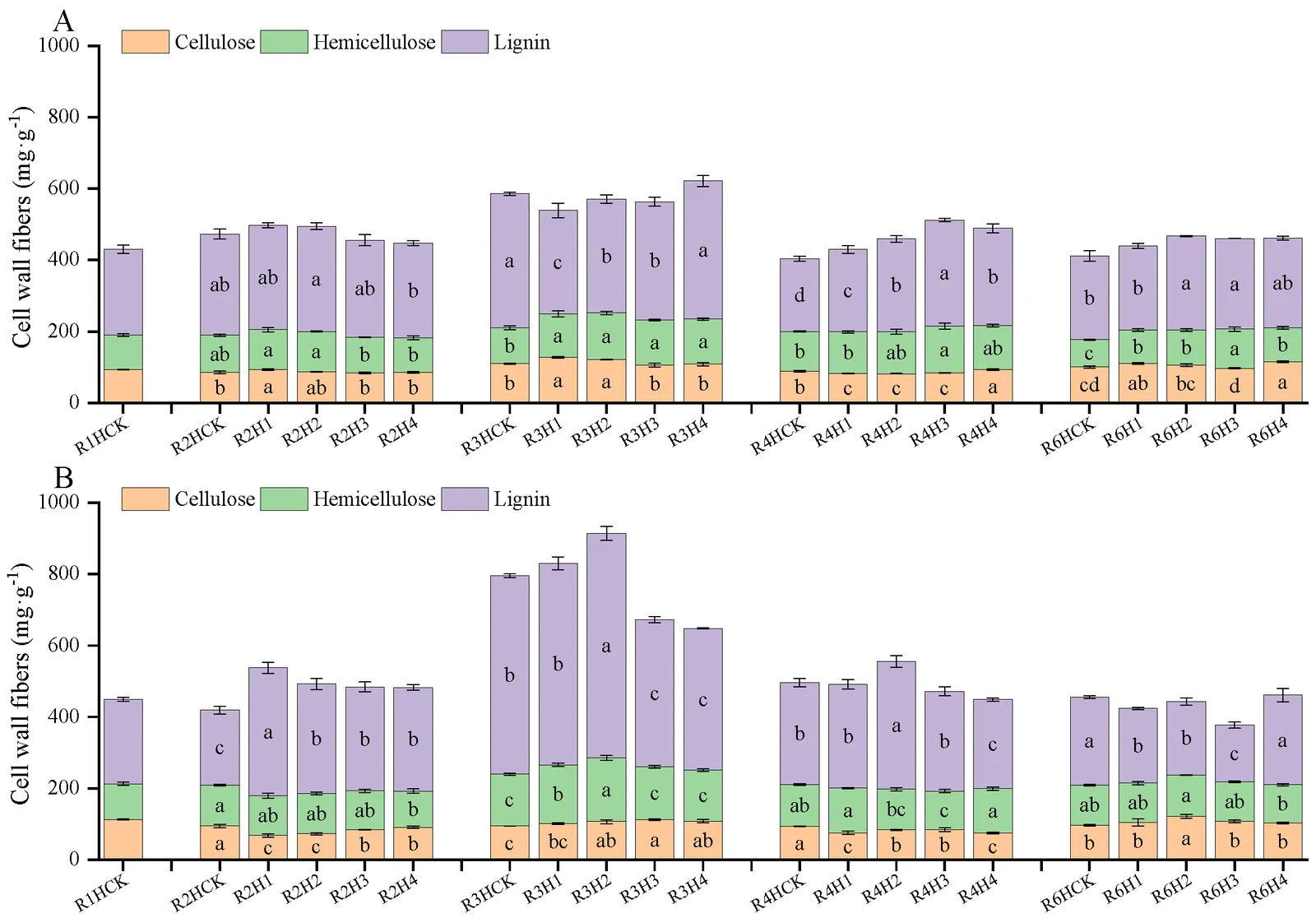
Effects of different MC concentrations applied at R1 on soybean stem cell wall components. (**A**), HN65; (**B**), HN44. R1MCK denotes the MCK treatment sampled at R1; R2MCK–R2M4, R3MCK–R3M4, R4MCK–R4M4, and R6MCK–R6M4 denote treatments MCK–M4 sampled at R2, R3, R4, and R6, respectively. MCK is the water-sprayed control (0 mg/L MC); M1, M2, M3, and M4 represent 50, 100, 200, and 400 mg/L MC, respectively. Different lowercase letters indicate significant differences among MC treatments for the same cell wall component within the same cultivar and sampling stage (*p* < 0.05).

As shown in Figure 7, R1 application of MC also significantly altered stem cell wall components in both cultivars, but the concentration producing the strongest response for cellulose, hemicellulose, and lignin varied with cultivar and sampling stage. As with V3 application, no simple linear concentration response was observed following R1 application.

The cellulose response of HN65 was relatively limited. At R2, M1 and M2 increased cellulose content by 9.06% and 2.20%, respectively, relative to MCK, whereas at R6, M1 and M4 increased it by 10.18% and 14.67%. In contrast, hemicellulose showed larger numerical changes and a more pronounced response to MC. At R3, M1, M2, M3, and M4 increased hemicellulose by 20.81%, 30.00%, 26.40%, and 25.24%, respectively; at R6, the corresponding increases were 22.34%, 28.50%, 42.62%, and 24.20%, with the largest increase under M3. At R4, lignin generally increased and then decreased as concentration increased; M1, M2, M3, and M4 increased lignin by 13.42%, 27.79%, 45.87%, and 33.98%, respectively.

Cell-wall components in HN44 also showed clear divergence. At R3, cellulose content under M1, M2, M3, and M4 was 7.13%, 13.04%, 18.77%, and 14.85% higher than MCK, respectively, and at R6 the largest increase occurred under M2, reaching 25.85%. At R3, hemicellulose responded most strongly to M2, increasing by 23.07% relative to MCK, whereas M4 caused a slight decrease. Lignin showed a strong response to lower concentrations at R2, with increases of 71.02%, 46.74%, 38.89%, and 38.41% under M1, M2, M3, and M4, respectively, relative to MCK.

### 2.8. Interactive Effects of Application Timing, Sampling Stage, and MC Concentration on Soybean Stem Traits

To further clarify the combined regulatory effects of application timing, sampling stage, and MC concentration on soybean stem traits, a three-way ANOVA was conducted separately for each trait in HN65 and HN44 (Table 1). For HN65, sampling stage and MC concentration significantly affected plant height, basal stem diameter, fifth-internode diameter, stem breaking force, bending deflection, and lignin, hemicellulose, and cellulose contents (*p* < 0.01). Application timing significantly affected plant height, basal stem diameter, stem breaking force, bending deflection, and hemicellulose content (*p* < 0.01), but its main effects on fifth-internode diameter, lignin, and cellulose contents were not significant (*p* > 0.05). The three-way interaction significantly affected basal stem diameter, stem breaking force, and all three cell-wall components, but not plant height, fifth-internode diameter, or bending deflection. For HN44, application timing, sampling stage, and MC concentration all showed significant main effects on every measured trait (*p* < 0.01). The two-way interactions were generally significant, and the three-way interaction of application timing × sampling stage × MC concentration was significant for all traits except basal stem diameter (*p* < 0.05).

### 2.9. Correlation Analysis Between Cell Wall Components and Stem Mechanical Properties Under MC Treatment

**Figure 8:**
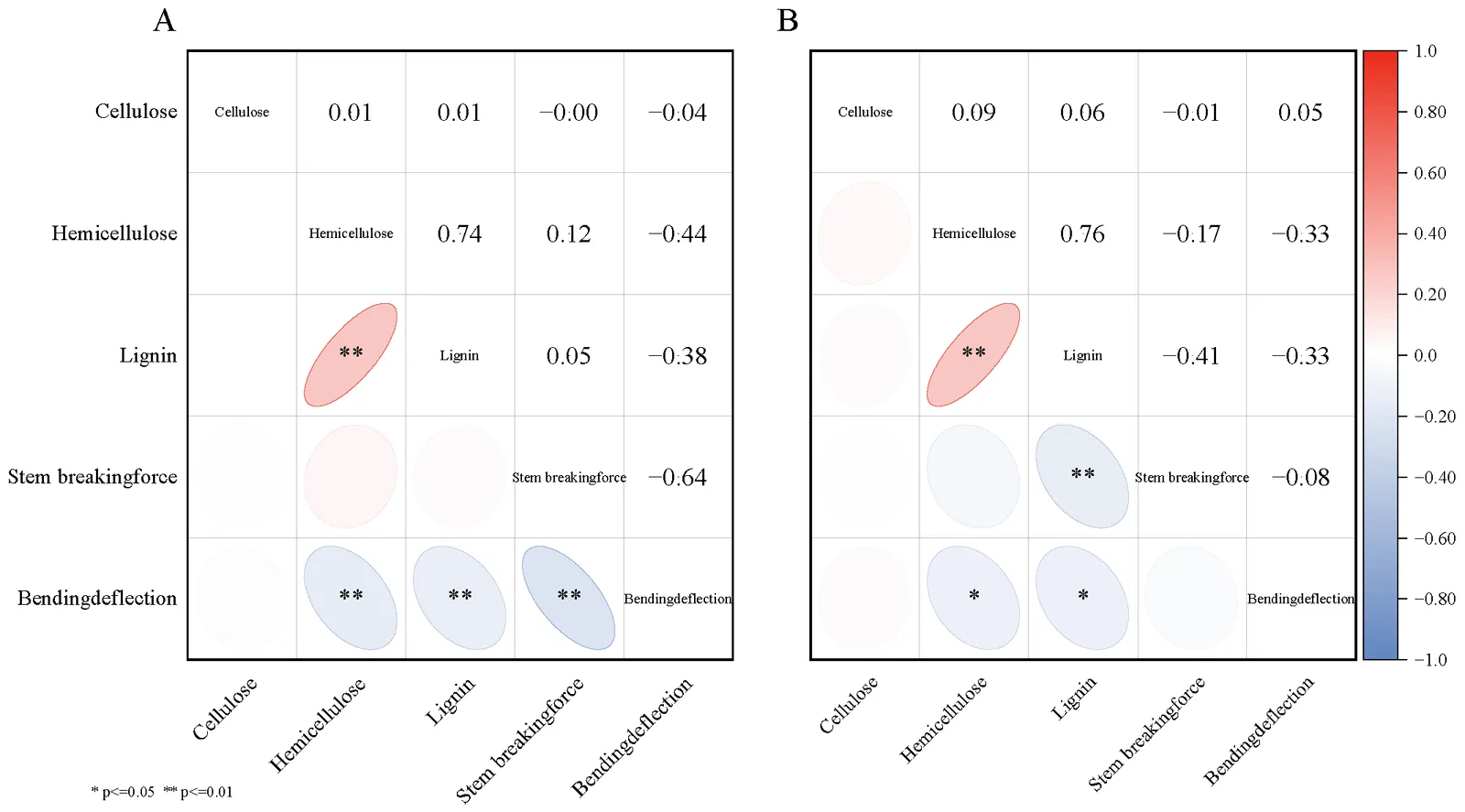
Correlation analysis between cell-wall components and stem mechanical properties following mepiquat chloride (MC) application. (**A**) Pearson correlation analysis of cellulose, hemicellulose, lignin, stem breaking force, and bending deflection following MC application at the V3 stage; (**B**) Pearson correlation analysis of the same variables following MC application at the R1 stage. Values represent Pearson correlation coefficients (r). Asterisks indicate statistical significance: * *p* < 0.05, ** *p* < 0.01.

To further examine the relationships between stem cell wall components and mechanical traits under different MC application stages, correlation analyses were conducted for cellulose, hemicellulose, lignin, stem breaking force, and bending deflection following V3 and R1 applications (Figure 8).

Following V3 application, cellulose was positively correlated with hemicellulose and lignin and negatively correlated with stem breaking force and bending deflection, although none of these correlations was significant. Hemicellulose was significantly positively correlated with lignin and was positively but not significantly correlated with stem breaking force; it was significantly negatively correlated with bending deflection. Lignin was positively but not significantly correlated with stem breaking force and significantly negatively correlated with bending deflection. In addition, stem breaking force was significantly negatively correlated with bending deflection.

Following R1 application, cellulose was positively correlated with hemicellulose, lignin, and bending deflection and negatively correlated with stem breaking force, but none of these relationships was significant. Hemicellulose was significantly positively correlated with lignin and negatively correlated with both stem breaking force and bending deflection, with the negative correlation with bending deflection reaching significance. Lignin was negatively correlated with both stem breaking force and bending deflection, and both negative correlations were significant. Stem breaking force was negatively correlated with bending deflection, but the relationship was not significant.

Overall, the directions of the relationships among cell-wall components and between cell-wall components and stem mechanical traits differed between V3 and R1 applications. Hemicellulose and lignin were significantly and positively correlated at both application timings, whereas some cell-wall components were significantly and negatively correlated with bending deflection or stem breaking force. These results suggest that changes in cell-wall components are not uniformly associated with changes in stem mechanical properties and that the correlation pattern depends on MC application timing.

## 3. Discussion

Plant height and stem diameter are important morphological traits that influence crop architecture and stem stability [22]. As a plant growth retardant, MC can inhibit cell elongation by modulating gibberellin-related growth processes, thereby restricting longitudinal plant growth [23]. Previous studies have shown that MC reduces GA3 and GA4 contents in elongating cotton internodes, regulates genes involved in GA biosynthesis and metabolism, and decreases the expression of GhEXP and GhXTH2, which are associated with cell-wall loosening and elongation, thereby inhibiting internode cell elongation [24]. In the present study, V3 application of MC suppressed plant height to varying degrees in both HN65 and HN44 during the early post-treatment period, with HN44 responding more strongly to 400 mg/L MC. However, this inhibitory effect gradually weakened as development progressed, and plant height under some treatments reached or exceeded the control during the middle and late growth stages. In contrast, R1 application did not result in sustained suppression of plant height, indicating that MC regulation is strongly dependent on application timing. Previous studies have likewise shown that MC can inhibit internode elongation and reduce plant height [23]. Cheng et al. [25] reported that, in soybean, MC significantly reduced plant height, shortened internodes, and increased stem diameter, thereby improving plant architecture and reducing lodging risk. These findings are generally consistent with the early suppression of plant height observed after V3 application in the present study. However, our results further showed that this inhibition was not sustained throughout development. As plants developed, differences in plant height among treatments gradually narrowed, and some treatments even reached or exceeded the control during the middle and late stages. This pattern may reflect the stage-dependent nature of MC regulation. As the interval after treatment increased, the effect of early MC application on the longitudinal elongation of newly formed tissues may have weakened, while subsequent growth may have involved recovery or compensatory elongation, thereby reducing the height differences established earlier. Stem diameter showed a response pattern distinct from that of plant height. Radial growth of soybean stems can continue after internode elongation has ceased [26], suggesting that MC regulation of longitudinal elongation and radial thickening is not synchronous. In the present study, appropriate MC concentrations increased both basal stem diameter and fifth-internode diameter at several growth stages, with a particularly pronounced response in HN44. This finding is broadly consistent with previous reports that MC reduces plant height while promoting stem thickening [27]. Thus, MC does not simply suppress growth; rather, it may rebalance longitudinal elongation and radial thickening. Furthermore, cultivar-specific differences in plant architecture responses to MC may be associated with inherent differences in lodging resistance-related traits. Previous studies by our research group showed that HN44 and HN65 exhibited distinct response patterns in stem breaking force, bending characteristics, and lodging resistance index following MC treatment [21]. The present study further showed that the two cultivars differed in their responses to MC in terms of plant height, stem diameter, stem mechanical properties, and cell-wall components, indicating strong cultivar dependence of MC regulation. These contrasting responses further support the use of HN44 and HN65 as suitable materials for comparing stage- and concentration-dependent effects of MC on plant architecture, stem mechanical properties, and cell-wall components.

Stem mechanical strength is an important determinant of resistance to stem lodging in crops [28,29]. The basal stem of soybean supports the load of the upper canopy and is exposed to external forces; therefore, its morphology and mechanical properties are closely associated with lodging resistance [30]. Previous studies have shown that soybean lodging resistance is closely related to stem breaking force, whereas greater plant height generally increases lodging risk [31,32]. Thus, reducing plant height, promoting stem thickening, and increasing stem mechanical strength may represent important pathways for improving soybean lodging-resistance potential. In maize, MC treatment has been reported to increase stalk bending strength and lignin accumulation [16]. In the present study, MC increased stem breaking force and bending deflection under specific combinations of cultivar, application stage, and concentration, indicating that MC can influence both resistance to fracture and bending deformation. In both cultivars, breaking force generally increased and then decreased with development and reached relatively high values at R4, suggesting that R4 may be an important stage for the formation and maintenance of stem mechanical strength. After V3 application, M3 increased stem breaking force of HN44 by 113.64% relative to MCK at R1; after R1 application, M4 increased stem breaking force of HN44 by 36.23% relative to MCK at R4. These results indicate that MC regulation of stem mechanical strength is not limited to the short period immediately after treatment but can persist into the middle and late growth stages. Bending deflection showed more pronounced stage- and concentration-dependent responses. After V3 treatment, bending deflection increased in both HN65 and HN44 at R1, with M4 producing increases of 27.01% and 24.46%, respectively, relative to MCK. After R1 treatment, responses of the two cultivars diverged further among concentrations. These results indicate that MC affects not only resistance to stem breakage but also the bending-deformation characteristics of stems under external loading [33]. The changes in mechanical properties induced by MC were broadly consistent with changes in stem morphology. Previous studies have shown that appropriate MC concentrations can increase basal stem diameter and fifth-internode diameter, and thicker stems usually provide a larger load-bearing cross-sectional area and greater structural stability, which may improve the ability of stems to withstand external forces [34]. Other studies have also shown that MC can improve crop lodging resistance by reducing plant height and increasing stem strength [35]. Therefore, MC-induced regulation of plant height, stem thickening, and enhanced mechanical performance may jointly constitute the morphological and mechanical basis for improved soybean stem stability. Three-way ANOVA further showed that stem breaking force and bending deflection were jointly regulated by application timing, sampling stage, MC concentration, and their interactions, confirming the clear timing- and cultivar-dependent nature of the MC response in soybean stem mechanical performance. Nevertheless, stem breaking force and bending deflection primarily describe the mechanical properties of the stem itself, whereas field lodging is also influenced by root anchorage, plant center of gravity, canopy load, and wind and rainfall conditions. Accordingly, the present results are more appropriately interpreted as evidence that MC regulates soybean stem mechanical stability and traits associated with stem-lodging resistance potential, rather than as direct evidence of improved whole-plant lodging resistance under field conditions.

Stem mechanical performance is also closely related to cell wall structure and composition. Lignin, cellulose, and hemicellulose are major structural components of secondary cell walls: lignin primarily contributes to wall reinforcement, cellulose forms the principal structural framework, and hemicellulose interacts with cellulose and lignin to help maintain cell wall structural stability [36,37]. Previous studies have shown that changes in stem cell-wall components are closely associated with stem rigidity, mechanical strength, and lodging resistance [38]. Therefore, MC-induced changes in cell-wall composition may provide an important structural basis for its regulation of soybean stem mechanical properties. In this study, MC applied at V3 significantly altered cellulose, hemicellulose, and lignin contents in the stems of both cultivars, although responses differed among components and cultivars. HN65 showed a relatively strong early cellulose response, with M2 increasing cellulose content by 36.50% relative to MCK at R1. HN44 showed a stronger lignin response, with M2 increasing lignin content by 77.39% relative to MCK at R2. After R1 application, M3 increased hemicellulose content of HN65 by 42.62% at R6 and lignin content by 45.87% at R4, whereas in HN44, M1 increased lignin content by 71.02% at R2. These findings indicate clear timing- and concentration-dependent responses of different cell-wall components to MC rather than a simple linear relationship in which higher concentrations lead to greater accumulation. These cell-wall changes were also broadly consistent with the stem mechanical responses described above. Appropriate MC concentrations increased stem breaking force and bending deflection under some treatments and were accompanied by increases in cellulose, hemicellulose, or lignin. Previous studies have indicated that higher cellulose content is associated with increased stem rigidity, while accumulation of hemicellulose and lignin also helps maintain cell-wall mechanical stability [39]. Related studies have further shown that carbohydrate accumulation in stems can affect lodging performance by influencing stem filling and mechanical strength [40,41]. Therefore, the increases in cell-wall components observed in this study may be associated with improved stem mechanical performance following MC treatment.

In addition to plant architecture and stem structure, MC can affect several physiological and biochemical processes. For example, Zhang et al. reported that MC treatment affected soluble protein content and the activities of protective enzymes such as SOD, POD, and CAT in cotton [42]. Another cotton study found that, under high-density and late-sowing conditions, higher MC doses reduced photosynthetic rate, Rubisco activity, and the activities of some amylases and were accompanied by yield reductions [43], indicating that the physiological effects of MC depend on concentration and cultivation environment. The present study focused on plant architecture, stem mechanical traits, and cell wall components and did not measure relative water content, electrolyte leakage, chlorophyll, antioxidant enzyme activities, or yield. Therefore, changes in stem mechanical properties cannot be used to infer whole-plant physiological stress resistance or yield stability. In addition, only two cultivars, HN44 and HN65, were evaluated under pot conditions at a single site and during a single growing season. The findings therefore require further validation across additional soybean genotypes and in multi-year, multi-location field experiments.

## 4. Materials and Methods

### 4.1. Plant Materials and Experimental Site

Soybean cultivars Heinong 44 (HN44) and Heinong 65 (HN65), both bred by the Soybean Research Institute of the Heilongjiang Academy of Agricultural Sciences, were used. The experiment was conducted at the experimental base of Northeast Agricultural University (45°44′ N, 126°43′ E). Soil was collected from the 0–20 cm plow layer of a field previously cropped with maize, and its basic physicochemical properties are shown in Table 2. Mepiquat chloride (MC; 98% active ingredient) was purchased from Hebei Guoxinnuonong Biotechnology Co., Ltd., China (product standard Q/GXNN 020-2017). Daily maximum, minimum, and mean temperatures and precipitation in Harbin from April to October 2025 are presented in Supplementary Table S1.

### 4.2. Experimental Design

A pot experiment was conducted using plastic containers with an upper diameter of 35 cm, a lower diameter of 22 cm, and a height of 30 cm. Six drainage holes (1 cm in diameter) were made in the bottom of each container and covered with mesh to ensure drainage. Each pot was filled with 16 kg of experimental soil and buried in an experimental trench, with the pot bottom approximately 30 cm below the soil surface. Adjacent pots were placed side by side, with 40 cm between rows.

Eight soybean seeds, 6.5–7.0 mm in diameter, were sown in each pot. Before sowing, seeds were coated with metalaxyl-M and fludioxonil following conventional agronomic practice. Each pot received 0.5 g potassium sulfate (50% K_2_O) and 1.0 g diammonium hydrogen phosphate (18% N and 46% P_2_O_5_) as basal fertilizer. After expansion of the first trifoliate leaf, seedlings were thinned to three uniformly growing plants per pot. MC was applied exclusively by foliar spraying. Except for MC application stage and concentration, all treatments received the same soil, basal fertilizer, and routine crop management to minimize the influence of non-treatment factors.

Two MC application timings were established: the seedling stage (V3) and the beginning flowering stage (R1). At each application timing, five concentration treatments were used: the water control MCK (0 mg/L) and four MC treatments, M1 (50 mg/L), M2 (100 mg/L), M3 (200 mg/L), and M4 (400 mg/L). Each treatment had three independent biological replicates. The first foliar application was performed at the designated growth stage, followed by a second application at the same concentration 3 d later. The treatment solution was sprayed uniformly to cover the plant leaves. Three independent biological replicates were established for each treatment at every sampling stage; thus, each cultivar × MC application stage × MC concentration × sampling stage combination consisted of three independent pots, with each pot serving as one experimental unit and one biological replicate (n = 3). The three plants within a pot were not treated as independent biological replicates. Independent pots were used at different sampling stages, and the same experimental unit was not sampled repeatedly. For destructive measurements, including stem mechanical properties and cell wall components, samples at each stage were collected from the corresponding independent replicate pots (Table 3).

### 4.3. Measurements and Methods

Measurements were conducted at the V3, R1, R2, R3, R4, R6, and R8 growth stages according to the requirements of each trait. The specific sampling stages for individual traits are indicated in the corresponding experimental design and result figures.

#### 4.3.1. Plant Morphological Traits

Plant height: The distance from the cotyledon scar to the main-stem growing point was measured using a tape measure.

Stem diameter: Basal stem diameter and fifth-internode diameter were measured using a vernier caliper. The segment from the cotyledon scar to the opposite unifoliate leaves was defined as the first node (internode), and the segment from the opposite unifoliate leaves to the first trifoliate leaf as the second node (internode), with subsequent nodes defined sequentially. Basal stem diameter and fifth-internode diameter were measured at the midpoint of the corresponding internodes.

#### 4.3.2. Stem Mechanical Properties

Stem breaking force: Measurements were performed using a stem mechanical tester. The basal portion of the stem was placed on two supports spaced 4 cm apart. A loading head connected to a force sensor was moved at a constant speed until stem failure occurred. The maximum force recorded by the sensor at breakage was defined as the stem breaking force. Measurements were taken from the basal region spanning the first to second internodes [44].

Stem bending deflection: A three-point bending test was performed using the same mechanical testing device. The stem base was placed on supports spaced 4 cm apart, and the loading head was moved at a constant speed until stem failure. The maximum displacement of the loading point relative to its initial position at breakage was recorded and defined as stem bending deflection [44].

#### 4.3.3. Determination of Cell-Wall Components

Cellulose, hemicellulose, and lignin contents were determined in the basal stem. Cell-wall material was first extracted. Approximately 0.03 g of sample was mixed thoroughly with 1 mL of 80% ethanol at room temperature and then incubated in a 90 °C water bath for 20 min. After cooling to room temperature, the sample was centrifuged at 6000× *g* for 10 min, and the supernatant was discarded. The resulting pellet was washed twice, alternately with 80% ethanol and acetone; after each wash, the sample was vortexed for 2 min and centrifuged at 6000× *g* for 10 min. The resulting pellet was regarded as the crude cell-wall fraction. Amylase was then added, and the sample was incubated for 15 h, centrifuged, and washed twice with distilled water. The material was dried at 65 °C to constant weight to obtain the cell-wall extract [45,46]. Cellulose, hemicellulose, and lignin contents were then determined spectrophotometrically at 620, 540, and 280 nm, respectively.

### 4.4. Statistical Analysis

All experimental data were analyzed using IBM SPSS Statistics 21.0 (IBM Corporation, Armonk, NY, USA) based on three independent biological replicates. Within each cultivar, application timing, and sampling stage, differences among MC concentrations were evaluated by one-way analysis of variance (ANOVA), followed by Duncan’s multiple range test for mean separation. Differences were considered significant at *p* < 0.05; different lowercase letters in the figures indicate significant differences among MC concentration treatments (*p* < 0.05). Three-way ANOVA was also performed separately for HN65 and HN44, with application timing, sampling stage, and MC concentration treated as fixed factors. Main effects and the interactions application timing × sampling stage, application timing × MC concentration, sampling stage × MC concentration, and application timing × sampling stage × MC concentration were evaluated for plant height, basal stem diameter, fifth-internode diameter, stem breaking force, bending deflection, and lignin, hemicellulose, and cellulose contents. To evaluate the relationships between cell-wall components and stem mechanical traits separately following MC application at the vegetative and flowering stages, Pearson correlation analyses were conducted for traits measured after V3 (vegetative) and R1 (beginning flowering) applications. All correlation tests were two-tailed, with *p* < 0.05 considered statistically significant. All figures were generated using OriginPro 2026.

## 5. Conclusions

This study showed that MC applied at different growth stages and concentrations significantly altered soybean plant architecture, stem mechanical properties, and cell-wall components, with responses depending on application timing, concentration, and cultivar. V3 application mainly reduced plant height during the early post-treatment period, whereas some concentrations promoted radial stem thickening at specific developmental stages. The reduction in plant height following R1 application was comparatively weak, although some treatments still increased basal stem diameter and fifth-internode diameter. Regarding stem mechanical properties, 50 mg/L MC applied at V3 increased HN65 stem breaking force by 40.34% relative to MCK at R1, whereas 400 mg/L MC increased bending deflection in HN65 and HN44 by 27.01% and 24.46%, respectively. When applied at R1, 400 mg/L MC increased HN44 stem breaking force by 36.23% at R4. MC treatment also altered cellulose, hemicellulose, and lignin contents; however, correlation analysis indicated that the relationships between cell-wall components and mechanical traits were not consistently linear. Overall, MC affected morphological, mechanical, and cell-wall traits associated with soybean stem stability, and V3 application produced a more pronounced early effect on plant architecture. Nevertheless, the present study does not support a single, universally optimal MC concentration across different soybean genotypes.

## Figures and Tables

**Table 1:** Three-way ANOVA of the effects of application timing, sampling stage, and MC concentration on soybean stem traits.

Cultivar	Trait	Application Timing		Sampling Stage		MC Concentration		Timing × Sampling		Timing × MC		Sampling × MC		Timing × Sampling × MC	
		F	*p*	F	*p*	F	*p*	F	*p*	F	*p*	F	*p*	F	*p*
HN65	Plant height	35.71	<0.01	627.23	<0.01	17.86	<0.01	6.37	<0.01	4.05	<0.01	6.63	<0.01	1.22	0.22
	Basal stem diameter	23.88	<0.01	577.38	<0.01	11.21	<0.01	5.21	<0.01	4.67	<0.01	6.10	<0.01	2.11	<0.01
	Fifth-internode diameter	1.79	0.17	164.23	<0.01	7.93	<0.01	2.16	0.03	4.14	<0.01	6.77	<0.01	1.37	0.12
	Stem breaking force	15.68	<0.01	1632.88	<0.01	5.22	<0.01	26.23	<0.01	3.77	<0.01	4.48	<0.01	5.62	<0.01
	Bending deflection	18.01	<0.01	55.45	<0.01	21.02	<0.01	5.95	<0.01	5.78	<0.01	5.55	<0.01	1.42	0.10
	Lignin	2.62	0.08	428.34	<0.01	20.64	<0.01	35.54	<0.01	31.90	<0.01	23.74	<0.01	17.71	<0.01
	Hemicellulose	34.68	<0.01	153.99	<0.01	39.53	<0.01	13.04	<0.01	15.96	<0.01	7.70	<0.01	3.84	<0.01
	Cellulose	1.84	0.16	305.75	<0.01	14.28	<0.01	6.60	<0.01	19.25	<0.01	18.45	<0.01	7.56	<0.01
HN44	Plant height	116.44	<0.01	271.70	<0.01	17.93	<0.01	4.89	<0.01	12.44	<0.01	2.45	<0.01	2.74	<0.01
	Basal stem diameter	7.60	<0.01	154.75	<0.01	15.00	<0.01	2.41	0.02	6.84	<0.01	5.51	<0.01	1.13	0.31
	Fifth-internode diameter	8.63	<0.01	210.42	<0.01	11.38	<0.01	7.21	<0.01	7.12	<0.01	5.98	<0.01	1.61	0.04
	Stem breaking force	26.54	<0.01	1876.84	<0.01	20.83	<0.01	19.68	<0.01	9.76	<0.01	13.09	<0.01	4.47	<0.01
	Bending deflection	5.50	<0.01	44.64	<0.01	11.87	<0.01	8.43	<0.01	4.21	<0.01	10.83	<0.01	5.15	<0.01
	Lignin	206.46	<0.01	1975.23	<0.01	86.12	<0.01	70.08	<0.01	47.70	<0.01	69.04	<0.01	31.24	<0.01
	Hemicellulose	7.71	<0.01	801.32	<0.01	10.43	<0.01	6.03	<0.01	15.74	<0.01	23.71	<0.01	7.56	<0.01
	Cellulose	5.70	<0.01	214.56	<0.01	18.08	<0.01	23.66	<0.01	2.62	<0.01	36.39	<0.01	7.79	<0.01

**Table 2:** Basic physicochemical properties of the experimental soil.

Total Nitrogen	Total Phosphorus	Total Potassium	Alkali-Hydrolyzable Nitrogen	Available Phosphorus	Available Potassium	Organic Matter	pH
(g/kg)	(g/kg)	(g/kg)	(mg/kg)	(mg/kg)	(mg/kg)	(g/kg)	
0.79	1.01	18.31	126.67	39.59	180.59	31.53	7.21

**Table 3:** Mepiquat chloride treatment design for soybean cultivars.

Treatment	Application Stage	MC Concentration (mg/L)	Number of Applications
MCK	V3/R1	0	2
M1	V3/R1	50	2
M2	V3/R1	100	2
M3	V3/R1	200	2
M4	V3/R1	400	2

## Data Availability

The original data presented in this study are included in the article.

## Supplementary Materials

The following supporting information can be downloaded at: https://www.mdpi.com/article/10.3390/plants15203126/s1, Table S1. Daily maximum, minimum, and mean air temperatures and precipitation in Harbin, China, from 1 April to 31 October 2025.
