# Supplementary material for: Effects of Mepiquat Chloride Application Timing and Concentration on Soybean Plant Architecture and Stem Mechanical Properties
Source: Plants (Basel). 2026 Oct 13;15(20):3126. doi: 10.3390/plants15203126 (PMC13708849; doi:10.3390/plants15203126)
Supplement: Supplementary file 1 [file plants-15-03126-s001.zip › plants-4584652-supplementary.pdf]

Supplementary Table S1

Daily maximum, minimum, and mean air temperatures and precipitation in Harbin, China, from 1 April to 31 October 2025.

Date	Maximum air temperature (°C)	Minimum air temperature (°C)	Mean air temperature (°C)	Precipitation (mm)
2025-04-01	10.7	-0.2	4.4	10.9
2025-04-02	8.7	1.0	3.7	1.6
2025-04-03	12.7	-2.8	4.7	0.0
2025-04-04	15.6	-0.7	7.7	0.0
2025-04-05	11.2	3.6	5.8	2.2
2025-04-06	13.6	1.0	6.3	NA
2025-04-07	14.4	0.0	7.7	NA
2025-04-08	16.6	3.7	10.5	0.0
2025-04-09	20.3	8.5	14.0	0.0
2025-04-10	19.4	8.0	12.3	NA
2025-04-11	22.4	5.3	13.5	NA
2025-04-12	19.0	11.2	14.8	NA
2025-04-13	12.8	9.3	11.4	0.2
2025-04-14	11.8	3.9	7.4	3.7
2025-04-15	13.1	4.7	8.0	1.5
2025-04-16	14.2	-1.9	7.8	0.0
2025-04-17	18.6	2.3	10.7	0.0
2025-04-18	14.0	5.2	7.7	2.0
2025-04-19	13.3	2.7	9.2	0.0
2025-04-20	20.4	4.5	11.8	0.0
2025-04-21	20.2	9.6	13.6	5.9
2025-04-22	14.2	4.4	8.5	10.3
2025-04-23	19.4	4.6	11.5	0.0
2025-04-24	12.1	4.9	7.2	0.3
2025-04-25	9.4	4.1	6.4	NA
2025-04-26	16.8	1.8	10.2	0.0
2025-04-27	13.3	6.2	9.2	NA
2025-04-28	13.8	0.7	7.4	0.0
2025-04-29	20.0	5.6	13.6	0.0
2025-04-30	18.6	6.6	11.4	0.0
2025-05-01	15.8	6.5	11.0	0.0
2025-05-02	17.9	3.4	11.4	0.0
2025-05-03	15.8	6.6	10.2	2.6
2025-05-04	14.5	3.3	9.2	0.2
2025-05-05	16.4	5.4	11.1	0.0
2025-05-06	16.9	5.0	10.5	0.3
2025-05-07	17.0	3.1	10.2	0.1
2025-05-08	15.0	3.7	9.6	1.0
2025-05-09	18.3	9.5	12.6	18.0
2025-05-10	20.2	7.8	13.6	0.0
2025-05-11	26.5	12.9	19.5	0.0
2025-05-12	25.9	17.0	20.9	0.0
2025-05-13	24.1	15.9	20.2	0.2
2025-05-14	23.1	13.3	16.8	NA
2025-05-15	22.3	8.4	16.3	NA
2025-05-16	19.3	7.9	10.2	2.0
2025-05-17	12.9	6.3	8.8	12.3
2025-05-18	17.3	5.8	11.8	0.0
2025-05-19	18.2	7.7	12.4	NA
2025-05-20	20.2	7.1	14.7	0.0
2025-05-21	20.7	6.3	14.2	0.0
2025-05-22	21.0	9.7	15.9	0.0
2025-05-23	23.5	7.3	17.8	0.0
2025-05-24	19.2	10.6	14.7	2.0
2025-05-25	23.3	8.9	17.5	NA
2025-05-26	23.8	12.6	17.6	8.6
2025-05-27	28.9	12.4	21.0	0.1
2025-05-28	29.8	13.6	22.4	NA
2025-05-29	29.8	16.2	24.2	NA
2025-05-30	31.2	14.4	24.9	NA
2025-05-31	28.4	19.1	24.3	0.0

Date	Maximum air temperature (°C)	Minimum air temperature (°C)	Mean air temperature (°C)	Precipitation (mm)
2025-06-01	23.8	14.2	17.0	NA
2025-06-02	18.3	10.1	15.0	9.2
2025-06-03	20.1	12.2	15.7	3.0
2025-06-04	23.8	10.1	17.7	0.2
2025-06-05	21.5	13.3	16.0	11.8
2025-06-06	20.9	11.6	16.6	NA
2025-06-07	23.1	12.3	17.5	0.7
2025-06-08	27.4	12.4	19.9	0.2
2025-06-09	21.8	12.5	15.9	0.3
2025-06-10	15.3	13.2	14.2	30.0
2025-06-11	27.1	13.9	19.4	0.0
2025-06-12	27.2	15.0	21.9	NA
2025-06-13	25.4	18.8	22.3	NA
2025-06-14	27.0	17.8	21.4	0.9
2025-06-15	28.3	17.5	23.1	0.0
2025-06-16	26.5	18.6	21.7	6.2
2025-06-17	23.8	16.0	19.3	NA
2025-06-18	30.0	11.9	22.9	0.0
2025-06-19	27.5	20.4	23.5	2.8
2025-06-20	24.7	17.3	19.9	13.5
2025-06-21	20.8	13.3	17.0	7.8
2025-06-22	30.6	16.4	22.7	0.1
2025-06-23	33.4	19.2	26.8	NA
2025-06-24	34.5	23.8	29.3	0.0
2025-06-25	34.2	22.9	28.7	0.0
2025-06-26	33.1	22.6	27.0	0.0
2025-06-27	33.5	22.4	28.2	0.0
2025-06-28	29.3	22.1	25.0	0.7
2025-06-29	29.4	22.2	25.4	2.7
2025-06-30	28.9	21.9	25.4	5.5
2025-07-01	27.5	22.9	24.6	6.6
2025-07-02	31.0	22.4	27.0	3.6
2025-07-03	31.0	24.0	27.3	NA
2025-07-04	32.6	23.2	27.7	NA
2025-07-05	29.1	22.3	24.2	38.8
2025-07-06	28.6	18.7	23.2	0.0
2025-07-07	28.7	18.7	24.2	0.0
2025-07-08	28.4	18.9	24.8	0.0
2025-07-09	31.0	22.9	26.5	NA
2025-07-10	32.7	22.2	27.2	NA
2025-07-11	33.5	25.8	30.2	NA
2025-07-12	30.5	24.1	27.4	5.7
2025-07-13	29.1	20.4	24.8	NA
2025-07-14	29.8	18.6	24.5	0.0
2025-07-15	28.9	19.7	24.8	NA
2025-07-16	27.9	22.2	24.7	NA
2025-07-17	29.7	21.1	23.8	30.2
2025-07-18	27.8	20.4	23.8	0.3
2025-07-19	29.9	21.0	25.9	NA
2025-07-20	27.2	23.0	25.3	1.0
2025-07-21	30.0	20.2	24.6	NA
2025-07-22	32.2	23.5	27.7	NA
2025-07-23	32.0	24.3	28.1	0.0
2025-07-24	29.9	25.5	27.5	0.0
2025-07-25	28.6	21.8	24.9	9.7
2025-07-26	26.4	22.9	24.6	0.3
2025-07-27	30.6	22.5	26.0	NA
2025-07-28	30.6	19.5	24.9	0.0
2025-07-29	28.0	20.1	23.6	12.4
2025-07-30	27.8	20.5	24.2	NA
2025-07-31	29.6	19.5	23.9	0.0
2025-08-01	30.5	19.5	24.7	0.0
2025-08-02	30.4	18.8	24.3	31.4
2025-08-03	25.9	20.4	22.7	73.6
2025-08-04	29.9	18.0	24.1	NA

Date	Maximum air temperature (°C)	Minimum air temperature (°C)	Mean air temperature (°C)	Precipitation (mm)
2025-08-05	28.2	20.0	NA	NA
2025-08-06	27.7	19.3	22.8	39.2
2025-08-07	26.6	18.3	21.7	0.0
2025-08-08	28.1	16.6	22.9	NA
2025-08-09	26.0	17.3	21.4	6.6
2025-08-10	23.0	16.3	19.9	0.1
2025-08-11	23.7	14.7	18.8	0.0
2025-08-12	26.0	16.3	21.2	1.6
2025-08-13	26.8	19.5	22.4	35.4
2025-08-14	28.2	18.4	22.3	5.0
2025-08-15	29.8	19.4	24.9	NA
2025-08-16	26.2	18.2	21.1	NA
2025-08-17	24.3	14.1	18.8	0.0
2025-08-18	21.8	13.4	18.5	NA
2025-08-19	26.8	16.8	21.9	21.8
2025-08-20	27.1	16.2	21.0	NA
2025-08-21	25.8	16.2	21.7	NA
2025-08-22	22.2	18.5	19.4	10.7
2025-08-23	29.1	17.1	23.2	0.0
2025-08-24	29.1	20.1	23.5	0.0
2025-08-25	27.4	18.0	22.1	NA
2025-08-26	25.8	17.5	20.6	4.1
2025-08-27	27.2	16.4	21.1	0.0
2025-08-28	23.1	17.6	20.4	24.7
2025-08-29	25.9	17.1	21.3	NA
2025-08-30	28.2	19.0	21.7	0.0
2025-08-31	27.4	17.5	22.0	17.9
2025-09-01	27.3	18.6	22.6	NA
2025-09-02	27.9	17.1	21.8	0.0
2025-09-03	29.2	17.9	24.0	NA
2025-09-04	30.1	21.3	24.9	NA
2025-09-05	29.0	18.3	23.7	0.0
2025-09-06	25.6	16.5	20.5	0.7
2025-09-07	23.7	13.5	18.5	NA
2025-09-08	22.5	13.1	17.4	0.0
2025-09-09	22.9	11.3	17.1	0.0
2025-09-10	24.1	11.1	17.2	0.0
2025-09-11	26.1	11.5	18.9	0.0
2025-09-12	26.3	18.6	22.6	NA
2025-09-13	26.8	12.6	19.4	0.0
2025-09-14	27.0	17.1	21.4	1.3
2025-09-15	22.3	13.0	17.8	0.0
2025-09-16	19.6	12.5	15.3	3.4
2025-09-17	19.7	7.1	12.8	NA
2025-09-18	22.6	5.8	14.4	NA
2025-09-19	23.9	7.9	17.1	0.0
2025-09-20	22.6	9.7	15.8	0.0
2025-09-21	22.4	7.3	14.9	NA
2025-09-22	24.0	8.2	15.5	0.0
2025-09-23	25.7	9.5	17.7	0.0
2025-09-24	19.5	14.3	16.1	10.4
2025-09-25	20.3	12.3	15.7	NA
2025-09-26	24.3	12.1	17.8	NA
2025-09-27	26.4	14.2	19.8	NA
2025-09-28	24.7	12.2	18.2	0.0
2025-09-29	23.0	5.5	14.5	0.0
2025-09-30	24.8	12.5	19.3	NA
2025-10-01	24.5	11.9	17.4	NA
2025-10-02	25.2	5.4	15.1	0.0
2025-10-03	27.7	13.3	21.1	NA
2025-10-04	23.3	12.4	17.0	NA
2025-10-05	15.2	3.5	10.6	0.0
2025-10-06	15.4	1.6	8.4	0.0
2025-10-07	19.8	3.2	11.6	0.0
2025-10-08	12.0	2.4	7.2	0.0

Date	Maximum air temperature (°C)	Minimum air temperature (°C)	Mean air temperature (°C)	Precipitation (mm)
2025-10-09	13.3	-1.6	5.2	0.0
2025-10-10	15.3	1.2	7.2	0.0
2025-10-11	16.0	2.9	8.9	0.0
2025-10-12	11.3	0.2	5.5	0.0
2025-10-13	11.4	2.0	6.1	NA
2025-10-14	10.1	3.0	6.3	0.3
2025-10-15	6.0	-0.2	1.6	18.3
2025-10-16	5.7	-1.5	1.1	0.0
2025-10-17	2.1	-1.2	0.8	2.7
2025-10-18	3.8	-4.3	-0.4	0.0
2025-10-19	2.3	-4.5	-0.6	1.5
2025-10-20	4.6	-2.6	0.2	NA
2025-10-21	7.6	-3.9	1.5	0.0
2025-10-22	8.9	-4.5	2.1	0.0
2025-10-23	6.3	-5.4	-0.1	0.0
2025-10-24	7.8	-5.5	1.5	0.0
2025-10-25	10.2	-1.1	3.6	0.0
2025-10-26	5.2	-2.8	0.8	NA
2025-10-27	1.1	-5.7	-1.7	0.0
2025-10-28	4.2	-5.5	-1.5	0.0
2025-10-29	9.4	-6.4	3.0	NA
2025-10-30	9.4	-2.2	2.1	0.0
2025-10-31	11.0	-0.7	3.2	0.0

Notes.NA indicate trace precipitation events, for which precipitation was observed but no measurable amount was recorded.
